# The quality of life of Croatian women after mastectomy: a cross-sectional single-center study

**DOI:** 10.1186/s12889-018-5929-0

**Published:** 2018-08-10

**Authors:** Stana Pačarić, Jozo Kristek, Jure Mirat, Goran Kondža, Tajana Turk, Nikolina Farčić, Želimir Orkić, Ana Nemčić

**Affiliations:** 1 Department of Surgery, Faculty of Medicine, University of Osijek, Osijek, Croatia; 2 Department of Surgery, University Hospital Osijek, Huttlerova 4, 31000 Osijek, Croatia; 3 Faculty of Medicine, University of Osijek, Cara Hadrijana 10/E, HR, 31000 Osijek, Croatia; 4 Department of Biophysics and Radiology, Faculty of Medicine, University of Osijek, Osijek, Croatia; 5 Department of Diagnostic and Interventional Radiology, University Hospital Osijek, Huttlerova 4, 31000 Osijek, Croatia; 6 Department of Nursing, Medical Ethics and Palliative Medicine, Faculty of Medicine, University of Osijek, Osijek, Croatia

**Keywords:** Breast cancer, Quality of life, Mastectomy, Satisfaction, Woman

## Abstract

**Background:**

Measuring the quality of life (QoL) of women with breast cancer is an important aspect of measuring treatment success. In Croatia, no QoL studies have been carried out with a focus on patients after mastectomy. The aim of this study was to examine QoL 1 month and 1 year after mastectomy.

**Methods:**

This cross-sectional single-center study of quality of life was conducted in 101 patients, 50 of whom had undergone a mastectomy 1 month prior, and 51 of whom had undergone a mastectomy 1 year prior. The study was conducted from July 2015 to June 2016. The questionnaires used in the study were developed by the European Organisation for Research and Treatment of Cancer (EORTC). The questionnaire EORTC QLQ-C30 assesses the QoL of cancer patients, and the questionnaire EORTC QLQ-BR23 is a disease-specific breast cancer module. A chi square test, Fisher’s exact test, Kolmogorov-Smirnov test, Student’s t-test and Mann-Whitney U test were performed in the statistical analysis using the statistical program SPSS (Inc. Released 2008. SPSS Statistics for Windows, Version 17.0. Chicago: SPSS Inc.).

**Results:**

Patients who had undergone a mastectomy a year earlier placed a higher value on their health state than did those who had undergone a mastectomy a month earlier. The most affected values of functional status on the EORTC QLQ-C30 scale were emotional functioning (37.5 [95% CI 33.3–61.6]) and sexual functioning (16.67 [95% CI 0–33.3]) 1 month and 1 year after mastectomy, respectively. The most affected symptoms on the EORTC QLQ-C30 scale were hair loss 66.67 [95% CI 33.3–100]) and fatigue 33.33 [95% CI 24–44]) 1 month and 1 year after mastectomy, respectively.

**Conclusion:**

In our study, both functional and symptom scales were more affected in women 1 month after mastectomy. QoL was considerably improved in women 1 year after the surgery compared to 1 month after mastectomy. The results of this study could contribute to the public awareness of the QoL of breast cancer patients.

## Background

Breast cancer is one of the most common malignant cancers, and it affects women all over the world [1]. In the Republic of Croatia, breast cancer is a significant public health problem and is the second leading cause of death after cardiovascular diseases. For the period between 2010 and 2014, the age-standardized incidence rate was 119.2 cases per 100,000 women, with a mortality rate of 48.3%. There are 2644 (26%) new cases every year [2]. In the world, as well as in Croatia, the number of cases of breast cancer has been increasing since the beginning of data collection on malignant diseases. For the period between 2009 and 2010, the data for Osijek-Baranya County are slightly higher than the Croatian average: 118 cases per 100.000 women per year [3]**.** The average life expectancy for women with breast cancer has risen significantly due to early diagnosis and new cancer treatments [4]**.** The average 5-year survival rate is 85%, whereas in developing countries, it is 50–60% [5]. Due to the increased survival of breast cancer patients, the impact of therapy on their quality of life (QoL) has become an important public health issue [6]. Although significant improvements have been made in recent decades regarding the detection and treatment of breast cancer, the disease still has a negative impact on social and physical functioning, especially in developing countries [7]. Only a study by Murgic et al. [8] has assessed the QoL in breast cancer patients in Croatia, comparing the QoL scores of patients receiving adjuvant treatment (adjuvant radiotherapy and adjuvant chemotherapy) with the QoL scores long-term breast cancer survivors.

Although no significant difference was found in QoL comparing these groups of patients, some scales of QoL were still more affected in long-term survivors, suggesting concomitant factors might contribute to overall QoL. No studies have been performed in Croatia with a focus primarily on patients after mastectomy. Quality-of-life measurements in women with breast cancer lately have become the focus of clinical practice and research, being significantly important in the assessment of treatment outcomes [9, 10]. According to the World Health Organization (WHO), quality of life is defined as “the individual’s perception of their position in life in the context of the culture and value systems in which they live and in relation to their goals” [11]. After mastectomy, women experience various functional and emotional disorders, such as depression, which leave serious psychosocial consequences. Some studies in western countries have shown that the prevalence of depression after mastectomy ranges from 1 to 56% [12]. Various treatments, such as surgical treatments, systemic therapy (chemotherapy, hormonal therapy, X-ray therapy and new targeted antibody therapy) and adjuvant endocrine therapy, affect the patient’s quality of life [13]**.**

Previous studies on quality of life, which assessed diagnosis, treatment, and healing, have shown that women with breast cancer are under increased risk of developing physical health problems (vomiting, sleep disorder and pain) and psychological distress (depression, anxiety, negative thoughts, fear of recurrence and death, feelings of being alone, sexual problems and poor self-image), all of which have a negative influence on quality of life and survival [14, 15].

### Objectives

The aim of this study was to examine the quality of life of women with breast cancer after a month and after a year from surgery and to compare the quality of life after a month and after a year from mastectomy.

## Methods

### Study design and setting

A cross-sectional study of quality of life in two female groups with breast cancer — after a month and after a year from surgery (mastectomy) — was conducted during the period from July 2015 to June 2016. The study was conducted at the University Hospital Osijek, which is a tertiary care hospital in Osijek-Baranya County, Croatia, with population of 305,000 inhabitants. The research was approved by the Hospital Ethics Committee (reference number 25–1: 11425–3/2015).

### Study population

The participants were enrolled during follow-up appointments at a thoracic surgery clinic.

The inclusion criteria were:

- women who had undergone a mastectomy (positive pathohistological finding of stage I or stage II breast cancer) 1 month earlier and were currently receiving adjuvant oncological therapy

The rationale for the chosen time point: patients have recovered from the surgery and are dealing with changes in QoL caused by the breast cancer diagnosis.

- women who had undergone a mastectomy (positive pathohistological finding of stage I or stage II breast cancer) 1 year earlier and were finished with adjuvant oncological therapy

Rationale for the chosen time point: patients have finished oncological therapy and are already accustomed to changes in QoL caused by the breast cancer diagnosis.

The exclusion criteria were age under 18 and/or over 75, a life expectancy less than a year, cognitive and/or mental diseases, illiteracy, and an inability to communicate in the Croatian language. Patients who were included in the study 1 month after mastectomy were not included again 1year later. There were 108 patients who met the criteria. During the study, 5 patients withdrew their approval to participate, and 2 questionnaires were not correctly completed; thus, the number of participants was decreased to 101 (93.5%). Before being asked to participate voluntarily, all participants were informed of the study’s objectives and assured of anonymity. Upon giving informed consent, the participants were also informed of the confidentiality of personal data and given the anonymous questionnaire, which they completed and handed to the researcher. Filling out the questionnaire took approximately 20 min.

### Study instruments

The questionnaires used in the research were developed by the European Organisation for Research and Treatment of Cancer (EORTC), and they were approved, translated into Croatian and validated by EORTC Quality of Life Group [16].

The questionnaire EORTC QLQ-C30 (version 3) was developed to assess the quality of life of cancer patients and consists of 30 items including five functional scales: physical, role, cognitive, emotional and social functioning; three symptom scales: fatigue, pain and nausea/vomiting; scales of the global health status/quality of life; and six individual items or symptoms usually associated with malignant disease: dyspnea, appetite loss, insomnia, constipation, diarrhea and financial difficulties following disease treatment. The scales and items are evaluated on a Likert scale of 4 levels, ranging from 1 (not at all) to 4 (almost always). A higher number of points correlates to poorer functioning and more symptoms. The exception is the global health status/quality of life scale; this is evaluated on a 7-point linear analogue scale, where a higher score indicates greater satisfaction with the global health status and quality of life [17].

The questionnaire EORTC QLQ-BR23 is a disease-specific module for breast cancer. It consists of 23 items and four functional scales: body image functioning, sexual functioning, sexuality, future health function; and a symptom scale consisting of the side effects of treatment, breast and arm symptoms and hair loss. The scales and items are evaluated on a Likert scale of 4 levels, ranging from 1 (not at all) to 4 (almost always). A higher score indicates poorer functioning [17].

All the results in the Likert scales (scores 1–4) and the linear analogue scale (1–7) were transformed into scores from 0 to 100 according to EORTC scoring. A higher score on the functional scale correlates to a higher (better) functional level, while a higher score on the symptom scale represents a higher (worse) level of symptoms [17].

Sociodemographic data, which includes age, education, and marital status, as well as clinical variables such as chemotherapy were collected during interviews.

### Statistical methods

To notice the effect of a 0.6 difference in numerical variables between the two independent participant groups, with a 0.05 difference in significance and a power of 0.8, the minimum size needed for the statistical sample was 45 participants per group, i.e., the total sample size was 90 participants. (This calculation was made using G*Power software version 3.1.2, written by Franz Faul, University of Kiel, Germany).

Categorical data are presented in absolute and relative frequencies. Numerical data are described by median and limits of the interquartile range (IC). Differences of categorical variables were determined by a chi square test and, if necessary, by Fisher’s exact test. The normality of the distribution of numerical variables was tested by the Kolmogorov-Smirnov test. Differences of numerical variables were tested by Student’s t-test and variables that deviate from normal distribution by the Mann-Whitney U test. All *P* values are two-sided. The level of significance was set at alpha = 0.05. The statistical program SPSS (Inc. Released 2008. SPSS Statistics for Windows, Version 17.0. Chicago: SPSS Inc.) was used for the statistical analysis.

## Results

### Sociodemographic characteristics

The study was conducted with 101 patients, 50 (49.5%) of whom had undergone surgery 1 month earlier and 51 (50.5%) of whom had undergone surgery 1 year earlier. The mean age of the patients was 56 years (standard deviation - 8.3 years) in the one-month post-surgery group and 54 years (standard deviation - 8.3 years) in the one-year post-surgery group (Table 1). All the patients were white females.

**Table 1:** The mean age of the patients and age upon the illness by groups

	Arithmetic mean (standard deviation)			*p* ^a^
	1 month after surgery	1 year after surgery	Total	
Age of the patients	56 (7.6)	55 (8.9)	56 (8.3)	0.660
Age at disease presentation	55 (7.6)	54 (8.8)	54 (8.3)	0.291

^a^Student’s t-test

Most of the patients (*n* = 49, 48.5%) had completed secondary education and 66 (65.3%) of them were married. Only 15 (14.9%) patients were living alone, and 73 (72.3%) of them considered the body image to be important and were familiar with the methods of reconstruction. There were 47 (46.5%) patients would have reconstruction if recommended by their surgeon. Within the sample, 44 (44%) patients started/underwent chemotherapy, 11 (11%) patients underwent radiotherapy, 41 (40%) patients had combined chemotherapy plus radiotherapy, and 5 (5%) patients were receiving hormonal therapy (Table 2).

**Table 2:** The main features of the patients by groups

	Number of patients (%)			*p* ^a^
	1 month after surgery	1 year after surgery	Total	
Level of education				
Unfinished primary school	1 (2)	2 (3.9)	3 (3)	0.331
Primary school	15 (30)	7 (13.7)	22 (21.8)	
High school degree	21 (42)	28 (54.9)	49 (48.5)	
College degree	7 (14)	6 (11.8)	13 (12.9)	
University degree	6 (12)	8 (15.7)	14 (13.9)	
Marital status				
Single	1 (2)	2 (3.9)	3 (3)	0.428
Married	33 (66)	33 (64.7)	66 (65.3)	
Common-law marriage	3 (6)	2 (3.9)	5 (5)	
Divorced	6 (12)	2 (3.9)	8 (7.9)	
Widowed	7 (14)	12 (23.5)	19 (18.8)	
Live alone	6 (12)	9 (17.6)	15 (14.9)	0.577^b^
Body image is important to them	35 (70)	38 (74.5)	73 (72.3)	0.661^b^
Familiar with the method of reconstruction	32 (64)	41 (80.4)	73 (72.3)	0.078^b^
Would agree to breast reconstruction if recommended by the surgeon	25 (50)	22 (43.1)	47 (46.5)	0.552^b^
Oncological therapy				
Chemotherapy	21 (42)	23 (45)	44 (44)	
Radiotherapy	5 (10)	6 (12)	11 (11)	0.96
Hormonal therapy	3 (6)	2 (4)	5 (5)	
Chemotherapy + Radiotherapy	21 (42)	20 (39)	41 (40)	
TOTAL	50 (100)	51 (100)	101 (100)	

^a^χ^2^ test; ^b^Fisher’s exact test

### Results for the EORTC QLQ-C30 questionnaire

The median values of functional status were significantly higher in patients 1 year after mastectomy (80 [95% CI 73.8–83.1]) compared to patients 1 month following mastectomy (57.78 [95% CI 48.9–68.5]). The most affected values of functional status on the EORTC QLQ-C30 scale were emotional functioning (37.5 [95% CI 33.3–61.6]) and sexual functioning (16.67 [95% CI 0–33.3]) 1 month and 1 year after mastectomy, respectively. The symptom scale was more affected 1 month after mastectomy (38.46 [95% CI 28.2–47.2]), compared to results 1 year following mastectomy (20.51 [95% CI 15.3–28.2]) The symptoms on the EORTC QLQ-C30 scale most often affecting patients were hair loss 66.67 [95% CI 33.3–100]) and fatigue 33.33 [95% CI 24–44]) 1 month and 1 year after mastectomy, respectively. There were no significant differences in insomnia, constipation, diarrhea and financial difficulties between the groups. All the results are presented in Table 3.

**Table 3:** Median values of the scale QLQ-C30 by groups of patients and in relation to the time elapsed after the surgery

QLQ-C30	Median (95% CI of median)			p*
	1 month after surgery**	1 year after surgery**	Total**	
Global health status	50 (33.3–53.3)	50 (50–66)	50 (50–58)	0.019
Physical functioning	73.33 (57.4–75.9)	86.67 (80–93)	80 (73.3–86.7)	0.003
Role functioning	66.67 (33.3–66.7)	83.33 (66.7–100)	66.67 (66–66.7)	0.003
Emotional functioning	37.5 (33.3–61.6)	66.67 (58.3–74.7)	58.33 (43.1–66.7)	0.014
Cognitive functioning	75 (66.7–83.3)	83.33 (83–100)	83.33 (83.3–83.3)	0.045
Social functioning	66.67 (50–83)	83.33 (83–100)	83.33 (66.7–83.3)	0.004
Functional scale total	57.78 (48.9–68.5)	80 (73.8–83.1)	73.33 (60.6–77.8)	0.002
Fatigue	55.56 (40–67)	33.33 (22–44)	44.44 (33.3–55.6)	0.039
Nausea/vomiting	16.67 (0–33)	0 (0–0)	0 (0–17)	0.001
Pain	41.67 (29–54)	16.67 (16.7–17)	16.67 (16.6–33.3)	0.014
Dyspnea	33.33 (33–67)	33.33 (0–33)	33.33 (33.3–33.3)	0.007
Insomnia	66.67 (33–67)	33.33 (33–66.7)	33.33 (33.3–66.6)	0.319
Appetite loss	33.33 (0–33)	0 (0–0)	0 (0–33.3)	0.004
Constipation	0 (0–33)	0 (0–33.3)	0 (0–33.3)	0.274
Diarrhea	0 (0–28)	0 (0–0)	0 (0–0)	0.497
Financial difficulties	33.33 (33.3–66.7)	33.33 (0–33)	33.33 (0–33.3)	0.139
Symptom scale total	38.46 (28.2–47.2)	20.51 (15.3–28.2)	28.21 (20.5–30.8)	0.007

*Mann-Whitney U test

**Higher score on the functional scale correlates to a higher (better) functional level; min: 0, max: 100

Higher score on the symptom scale represents a higher (worse) level of symptoms; min: 0, max: 100

### Results for the EORTC QLQ-BR23 questionnaire

The QoL evaluated by the disease-specific scale QLQ-BR23 showed no significant differences between the two groups regarding body image functioning (62,5 [95% CI 33,3-77,1] vs. 66,67 [95% CI 66,6-83,1] 1 month and 1 year after mastectomy, respectively) and anxiety over hair loss (66,67 [95% CI 33,3–100] vs. 33,33 [95% CI 11,1-33,3] 1 month and 1 year after mastectomy, respectively); these were the most affected items on the functional scale and the symptom scale, respectively. The functional scale in total was significantly more affected 1 year after the surgery (38.1 [95% CI 20.8–58.3] vs. 54.17 [95% CI 39.8–56.1]) 1 month and 1 year after mastectomy, respectively (*p* = 0.006) compared with the symptom scale 1 month after the surgery (36.75 [95% CI 25.4–63.8] vs. 21.43 [95% CI 17.8–35.6]) 1 month and 1 year after mastectomy, respectively, *p* < 0.001). All the results are presented in Table 4.

**Table 4:** Median values for the scales of the QLQ-BR23 by groups of patients and in relation to the time elapsed after the surgery

QLQ-BR23	Median (95% CI of median)					p*
	N	1 month after surgery**	N	1 year after surgery**	Total**	
Body image functioning	50	62.5 (33.3–77.1)	51	66.67 (66.6–83.1)	66.67 (58.3–72.7)	0.106
Sexual functioning	50	16.67 (0–16.7)	49	16.67 (0–33.3)	16.67 (0–16.7)	0.388
Sexual enjoyment	34	0 (0–33.3)	29	33.33 (0–33.3)	33.33 (8.3–33.3)	0.008
Future health function	49	0 (0–100)	51	33.33 (0–100)	33.33 (33.3–33.3)	0.003
Functional scale total	50	38.1 (20.8–58.3)	51	54.17 (39.8–56.1)	50 (37.5–54.2)	0.006
Systemic therapy side effects	50	38.1 (23.8–47.6)	51	19.05 (14.3–26.4)	23.81 (19.04–33.3)	0.001
Breast symptoms	50	33.33 (25–50)	51	16.67 (16.7–25)	25 (16.7–33.3)	0.008
Arm symptoms	50	44.44 (33.3–55.6)	51	22.22 (11.1–33.3)	33.33 (22.2–44.4)	0.001
Hair loss	26	66.67 (33.3–100)	22	33.33 (11.1–33.3)	66.67 (33.3–66.7)	0.057
Symptom scale total	50	36.75 (25.4–63.8)	51	21.43 (17.8–35.6)	26.67 (27.1–44.0)	< 0.001

*Mann-Whitney U test

**Higher score on the functional scale correlates to a higher (better) functional level; min: 0, max: 100

Higher score on the symptom scale represents a higher (worse) level of symptoms; min: 0, max: 100

## Discussion

The purpose of this study was to assess the QoL of Croatian breast cancer patients in order to obtain results that could be used to raise public awareness on this issue, as well as to plan and perform educational and interventional programs for more effective support. In this study, we used questionnaires that represent a standard for the evaluation of the QoL of breast cancer patients [16]. Although there is a certain overlap between the two questionnaires, the EORTC QLQ-C30 focuses on the general QoL of cancer patients, while the questionnaire EORTC QLQ-BR23 is a disease-specific module for breast cancer and, therefore, is more focused on the disease-specific factors that contribute to QoL.

### Functional scale for the EORTC QLQ-C30 questionnaire

The patients scored their QoL on the EORTC QLQ-C30 functional scale high, regarding body, professional, cognitive and social functioning, regardless of the period analyzed. This indicated that the level of functioning was satisfactory. Nevertheless, according to our results, the patients valued their health state higher 1 year after mastectomy compared to patients who had their mastectomy a month previous. These results are similar to the Polish QoL study on patients within a year from mastectomy [18]. Most of the participants were not confined to bed and needed no help with daily activities, including dressing, bathing and eating. They were also able to continue with their free-time activities and had no problems with concentration or memory [19]. Reduced emotional functioning was noticed in all patients. Our results were similar to the results from other studies, i.e., the results indicated the presence of frustration, anger, depression or anxiety [19–21]. Physical appearance and body image were important, especially for younger women who lost their breast due to mastectomy. The depression that these women might experience could lead to reduced quality of life regarding social interactions, mental health, and emotional functioning [22, 23]. Emotional support was effective for women with breast cancer since it provided an opportunity to express their feelings and needs and to share their experience about the disease. It has been noted that, over time, women may perceive a decrease in emotional support, which may be related to personality factors or to one’s psychological state [24]. Difficulties in emotional functioning influenced quality of life, especially during initial treatment when patients experienced the symptoms following adjuvant chemo-, radio- or hormonal therapy.

### Symptom scale for the EORTC QLQ-C30 questionnaire

For those patients who had undergone surgery a month earlier, the most common symptoms on the EORTC QLQ-C30 scale were fatigue, nausea/vomiting, pain, dyspnea, and appetite loss. Fatigue was the most common, yet underestimated, side effect of cancer treatment for breast cancer patients. It is a disturbing, persistent and subjective feeling of physical, emotional and/or cognitive weakness related to cancerous disease. This symptom worsens during radiation therapy and even more during chemotherapy. Anemia was also a contributing factor of fatigue [25]. Different interventional strategies are needed in order to solve these problems, and interventions should be tailored to each patient’s specific needs [26]. Chronic pain was the main clinical problem that affected 25 to 60% of patients. Developing chronic pain after breast cancer treatment, as well as after other surgical procedures, includes a complex pathophysiology consisting of pre-, intra- and postoperative factors [27]. The results showed that these painful symptoms could last for many years after mastectomy, thus decreasing the postoperative quality of life [28–31]. The results of a similar study conducted in Saudi Arabia that included 145 participants also showed disturbing symptoms such as insomnia, appetite loss and dyspnea [32].

### Symptom scale for the EORTC QLQ-BR23 questionnaire

According to the results of the EORTC QLQ-BR23 questionnaire, patients who had a mastectomy a month previous were most affected by the side effects of systemic therapy, arm symptoms, breast symptoms and hair loss-associated discomfort. Previously, published studies had shown similar results [20, 21, 33]. In our study, all of these symptoms had significantly diminished 1 year after mastectomy, except for hair loss. Hair loss was often seen as a prominent side effect of chemotherapy. Alopecia has a negative impact on body image and psychosocial well-being, especially in women, and is a major cause of depression in breast cancer patients [34].

### Functional scale for the EORTC QLQ-BR23 questionnaire

According to our study, women value their overall functioning better a year after mastectomy compared to functioning a month after surgery. These results were noted in both general oncologic and disease-specific questionnaires. A month after surgery, patients expressed the most problems regarding sexual functioning and enjoyment, as well as concern regarding future health functioning. One year following the mastectomy, there was no significant improvement regarding sexual functioning. Sexual dysfunction might occur as a consequence of premature menopause after adjuvant endocrine therapy in breast cancer patients [35]. The results of the previous study [36] showed that breast cancer patients after mastectomy felt not only less attractive, but they also disliked their physical appearance and felt incomplete. This might cause patients to feel insecure and avoid social interactions. Women after mastectomy also experience more problems with sexual desire, arousal and orgasm, which leads to sexual disfunction [37, 38].

### Limitations

The limitations of this study were its cross-sectional design as well as the small sample of participants.

There are also a variety of concomitant factors, an assessment of which was beyond the scope of this study, but that might influence the QoL of breast cancer patients, such as age, stage of disease at presentation, performance score of the patients, socioeconomic status, cultural values, spirituality, ethnicity, etc. [39–43]. Despite these limitations, the concept of disease-specific QoL questionnaires has been widely accepted and validated for their prognostic value in a clinical setting [44].

## Conclusion

The study results showed that breast cancer has a significant negative impact on the quality of life of breast cancer patients. Both functional and symptom scales were more affected in women 1 month after mastectomy. The QoL was considerably improved in women 1 year after mastectomy compared to those at 1 month.

The results of this study could contribute to the public awareness of the QoL of breast cancer patients and could also be of use in planning and performing educational and interventional programs for more effective support. Due to the variety of problems these patients encounter, a multidisciplinary approach is warranted for successful improvement of the QoL of breast cancer patients.

## Abbreviations

EORTC QLQ-BR23: European organization for research and treatment of cancer quality of life breast cancer questionnaire
EORTC QLQ-C30 (version 3): European organization for research and treatment of cancer quality questionnaire-cancer 30
EORTC: European organization for research and treatment of cancer
QoL: quality of life
